# Helicity Distributions of Single-Walled Carbon Nanotubes and Its Implication on the Growth Mechanism

**DOI:** 10.3390/ma3042725

**Published:** 2010-04-14

**Authors:** Sithara S. Wijeratne, Nolan C. Harris, Ching-Hwa Kiang

**Affiliations:** Department of Physics and Astronomy, Rice University, Houston, TX 77005, USA; E-Mails: ssw6@rice.edu (S.S.W.); nolanh@rice.edu (N.C.H.)

**Keywords:** carbon nanotube, helicity, growth mechanism

## Abstract

Single-walled nanotubes (SWNT) have attracted significant attention because of the substance’s superior crystal quality, high thermal conductivity and current carrying capacity, thus emerging as an attractive material for nanoelectrics. To optimize the selection of SWNT structures in large-scale synthesis, an understanding of their growth mechanism is necessary. We report studies of the helicity distributions of SWNT using electron nanodiffraction. The overall statistical distribution of helicity has peaks at 0° and 30°. The peak evident at 0° was found to be a sharp local maximum, while the peak at 30° was broader. We also found that the helicity distribution varies from region to region of micrometer size. This observation indicates that local environment affects nanotube growth, resulting in different structural distributions.

## 1. Introduction

The unique physical properties and potential applications of single-walled carbon nanotubes (SWNT) have been well documented since their discovery, using both theory and experiment [1,2,3,4,5,6]. It is evident that the useful electronic properties of SWNT are largely dependent on the atomic structures of individual nanotubes, which can be accurately defined using the tube diameter *d* and helical angle *θ* [7]. The significance that structure bears on the electrical properties of a nanotube is illustrated by the fact that a carbon nanotube’s ability to behave as either a metallic conductor or a semiconductor is determined by its *d* and *θ*. Combining SWNT of different electronic character to form macroscopic assemblies with physical properties of significant technological potential has been a major scientific goal [8,9]. With advances in synthesis techniques it has become possible to produce SWNT in large quantities at high yields. Although it has been suggested that SWNT *d* and *θ* are constant throughout the sample [10,11], experimental evidence has established that SWNT samples consist of isolated tubes and bundles of tubes varying in both these structural aspects [12,13,14].

**Figure 1:**
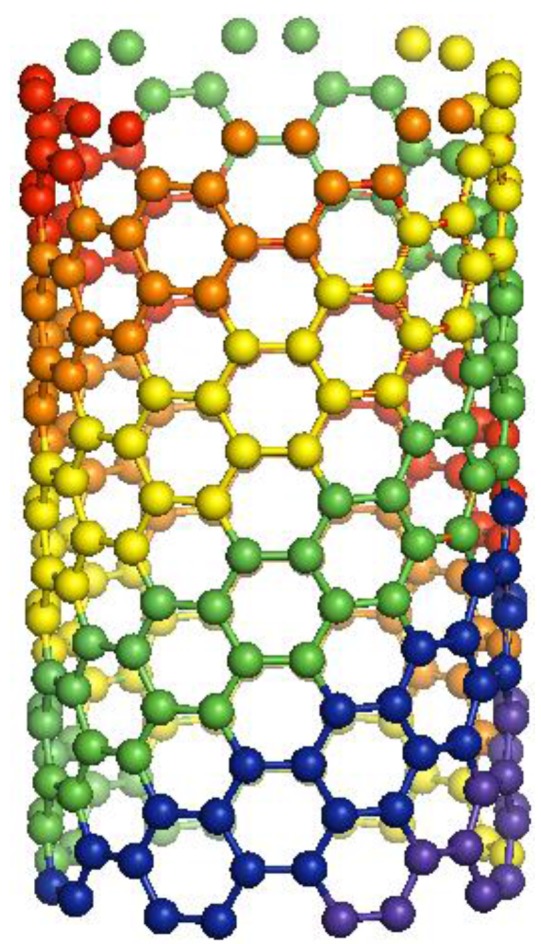
The helical structure single-walled carbon nanotubes (SWNT) depend on the nucleation and growth processes. Adding carbon atoms to an arm-chair structured SWNT is mechanistically different from to a zigzag structured tube. The illustration indicates adding carbon atoms to an arm-chair structured SWNT, and the color highlights the zigzag structure growing edges as a guide to the eye.

Many attempts have been made on separating SWNT of different structures [15,16,17], however, an effective method of separating SWNT in bulk is still lacking. Since the electrical [18,19,20,21,22,23] and optical [24,25,26,27,28,29] properties of SWNT depend on its structure, it is important to characterize the distribution of helicity of nanotube produced. Furthermore, a study of the *θ* distribution is important to understanding the growth of SWNT, as the process determines the final product of the helical structure of SWNT (see Figure 1). Uncovering the growth mechanism is essential, as the ultimate goal of nanotube synthesis remains producing SWNT of a specific structure.

**Figure 2:**
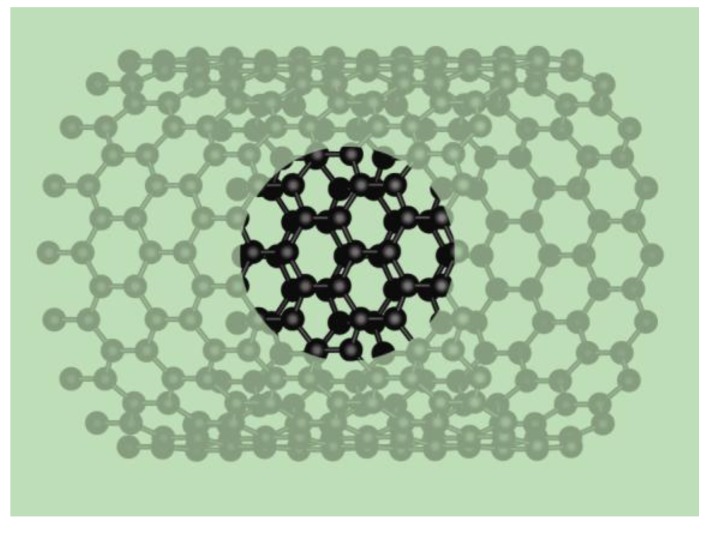
Illustration of the approximate spot size for convergent beam electron diffraction of a zigzag single-walled carbon nanotube. The spots are from a single tube, for which the helical angle *θ* can be determined unambiguously (see text).

In this paper, we present experimental studies of both the global and regional helicity distributions of both isolated and bundled SWNT. We analyzed the distribution of *θ* and *d* of SWNT produced with the arc discharge method using Co catalyst and various catalyst promoters [1,30,31,32]. We measured nanotube *θ* and *d* distributions in regions of one micrometer in diameter, using convergent beam electron diffraction (CBED), which produces diffraction patterns from regions of the sample that are 1 nm or less in diameter, allowing us to investigate small sections of nanotubes (see Figure 2) [33,34]. CBED allows us to study, with reduced beam damage, the individual helicity of tubes within a bundle, as well as helicity distributions of tubes from regions of micrometer size. However, CBED is limited by its resolution and accuracy, so a precise assignment of nanotube index is not possible [34]. A detailed description of the CBED technique can be found in Refs. [11,33,35]. Histograms of *θ* distributions for isolated *versus* bundled nanotubes, as well as for different regions, are compared. The results not only gave us a clear picture of the helicity distributions of the entire sample, but also provided insight into the mechanism of SWNT growth.

##  2. Results and Discussion

**Figure 3:**
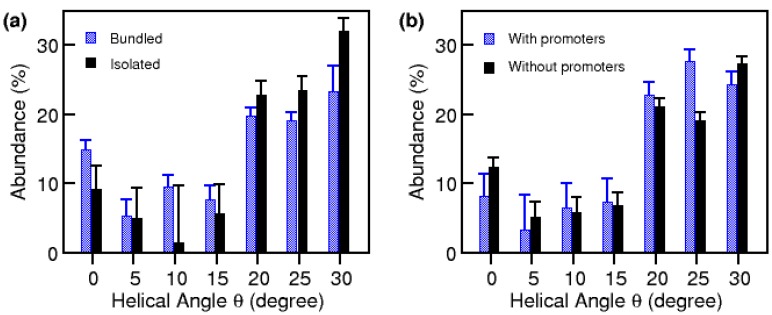
Helical angle distributions of SWNT. (a) Histograms of 140 isolated and 167 bundles of SWNT showing similar distributions, with a major peak at *θ =* 30*°* (armchair structure) and a minor peak at *θ* = 0*°* (zigzag structure), suggesting a similar growth mechanism for isolated and bundled SWNT. (b) Histograms of 307 SWNT produced by Co catalyst without promoters and 123 SWNT produced by Co catalyst and S, Pb, or Bi catalyst promoters, showing similar distributions. SWNT produced without catalyst promoters have a more distinct minor peak at near *θ* = 0*°.* The error bar is $\sqrt{N}$, where *N* is the number of samples in a given bin.

We recorded an average of twenty-five diffraction patterns each in eight separate regions, defined as SWNT within one micrometer in diameter. These experimental diffraction patterns were compared with calculated patterns to resolve the helicity of individual SWNT and bundles within each region, producing viable statistical distributions as to the regional helicity of SWNT, as well as over the entire sample. The diffraction window, as illustrated in Figure 2, was scanned across isolated tubes and individual bundles. When looking at bundles, we examined one tube on one bundle at a time and moved on to the next tube. Each diffraction pattern had a signal from vertically stacked tubes within one bundle. We found that helicity is mostly consistent within each bundle, but differs from bundle to bundle. However, occasionally diffuse diffraction spots were observed, indicating a spread of helical angles within a bundle, or possibly the presence of large amounts of defects in the SWNT structure. We also discovered that the overall distribution of *θ* for the sample, shown in Figure 3a, has a broad peak at 30° helicity (armchair structure). For the diffraction patterns recorded, more than 70% of the SWNT were found to have *θ* between 20° and 30°; more precisely, about 26% were verified to have *θ* of 30°, while 23% and 21% had *θ* of 25°and 20°, respectively. We also observed a local maximum at the *θ* = 0°, with 11% of the SWNT recorded showing the zigzag structure.

It is interesting to note that, for nanotubes produced with catalyst promoters, the distribution has a major peak at *θ =* 25°−30° and a less pronounced minor peak at *θ =* 0°, as shown in Figure 3b, indicating that the addition of catalyst promoters not only increases the nanotube production efficiency, but also modifies the diameter and helicity distributions of SWNT. The results indicate that the promoters assist the nanotube growth, preferentially stabilizing the nucleation of the non-zigzag SWNT [36]. The abundance of SWNT with helicity close to the armchair structure is in agreement with theories that predict that the growth rate increases essentially linearly with helicity angle [37,38,39].

**Figure 4:**
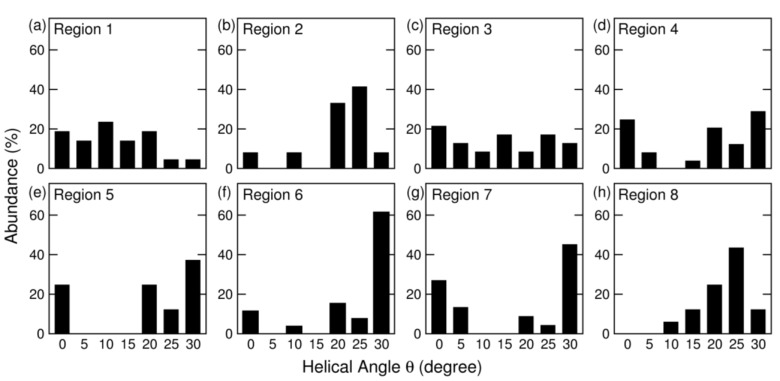
(a)-(h) Helical angle distributions of SWNT from 8 different regions of micrometer size, each containing 16−26 samples of nanotubes. Many of the distributions show a dominate peak at 30*°*; however, some of them have a uniform distribution of angles, indicating that difference in local environment influences the final SWNT product structure distribution.

The most striking feature of *θ* distributions is that the distributions are different for different micro regions. We determined the distributions of *θ* of regions of one micrometer in diameter and at least 5 micrometers apart, and compared the histograms from eight different regions. We discovered that the helicity distributions [40] varied from region to region, as shown in Figure 4. Some regions, such as region 6 was characterized by a single, large peak, greater than 55%, at *θ* = 30°, while other regions, such as region 3, demonstrated an almost uniform distribution of *θ* from 0° to 30°. This wide variation in distributions of *θ* suggests that SWNT growth, by carbon gas phase self-assembly, must be affected by the local environment at the time of growth.

**Figure 5:**
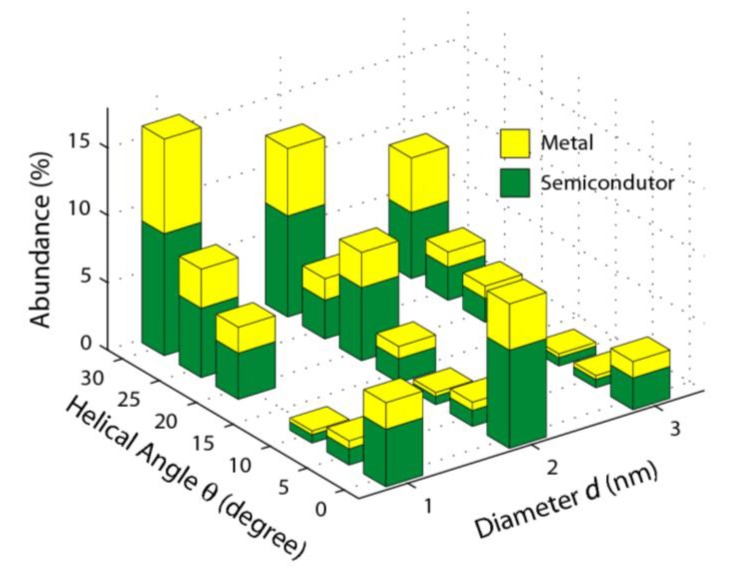
Distribution of SWNT helical angle *θ* and diameter *d* from isolated nanotubes. There is no apparent correlation between *θ* and *d*. The expected ratios between metallic and semiconducting nanotubes for each *θ* and *d* bin are shown.

There is no apparent relationship between the tube diameter *d* and helicity angle *θ*, as shown in Figure 5. Generally speaking, these results are consistent with the distributions of helical angles of double-walled carbon nanotubes, where the distribution of *θ* has a peak at 20° − 30° for *d <* 3 nm, and a more even distribution for *d >* 3 nm [41]. The distribution obtained from a different technique, nanobeam electron diffraction, also shows a preference for SWNT helicity at 15°−30° [42]. The broad peak in at 20° − 30° probably explains why, in early experiments, it was suggested that SWNT likely had uniform helicity throughout [10]. Note that nanotubes of *θ =* 30° are predicted to have metallic behavior, while those with *θ =* 0° may act as either metals or semiconductors, depending on *d*.

Furthermore, it has been predicted that the lower and upper bounds of stiffness for tubules with similar *d* are set by the *θ =* 0° and 30°, respectively. Using the method described in [43,44], we estimated that about 1/3 of the nanotubes are metallic [45]. Characterization of the distribution of *θ* and *d* in bulk SWNT samples provides valuable information on the distribution of physical properties that can be expected from a high yield, large scale synthesis of SWNT.

One proposed theory, the polyyne ring nucleus growth (PRN) model [39,46,47], addresses the importance of local environment in the growth of vapor-grown SWNT. Briefly, the PRN model asserts that the presence of electrons and catalyst particles in the arc plasma may, at high temperatures, cause the uniform planar form of a monocyclic ring to deform, producing local *cis* (armchair) or *trans* (zigzag) carbon conformations. The *cis* and *trans* conformations function as building blocks, by which to add C_2_ or C_3_, respectively. For example, adding one C_2_ species to the *cis* conformation will result in a non-helical armchair tubule. Helical angles are thus determined by the ratio of *cis* to *trans* conformations of the nucleus during growth. Therefore, the initial concentration of C_2_ and C_3_ species in the arc plasma may also affect the ratio of zigzag *versus* armchair structured SWNT. It is known that in the arc, C_3_ is more abundant than C_2_, thus in favor of the nucleation of zigzag structured SWNT. The PRN model provides a realistic mechanism to explain the diversity in SWNT growth from region to region by considering nanotube growth within a local environment.

The helicity of SWNT is determined by several factors, and both nucleation and growth processes are affected by environmental parameters such as the existence of catalysts and promoters, as well as local temperatures. Catalysts and promoters may affect the stability of different carbon cluster conformations, which determine the diameter and helicity distributions of the SWNT. For example, growth of SWNT is expected to favor the armchair *versus* the zigzag structures both thermodynamically and kinetically [37,38,39], but higher temperature will make the kinetic effect less important in determining the final helicity distributions. Therefore, the uniformly higher abundance of armchair structured tubes observed in samples produced in relatively low temperatures such as chemical vapor deposition (CVD) is consistent with the theory. The arc-discharge method, however, is capable of providing a higher temperature environment, and the linear dependence may not be as a dominating effect [40]. The temperature in the arc plasma is estimated to be around 3000−4000K [37]. At such high temperatures, the kinetic barrier is more easily overcome, making the linear dependence of the helicity distribution not as evident, which may explain the near uniform distributions of SWNT helical angles observed in regions 1 and 3. Note that the helicity distribution of arc discharge method is mainly for double-walled carbon nanotubes [41], which may have additional factors affecting the distributions. In addition, our histogram has higher resolution, which can resolve the minor peak at near *θ =* 0°, which is consistent with that determined by nanobeam electron diffraction [42].

## 3. Experimental Section

SWNT samples were produced using an arc discharge technique [1,30,31]. In brief, an electric arc was generated between graphite electrodes, of which the anode contains a mixture of graphite and transition metal catalyst, under a helium atmosphere. A cobalt based catalyst was used, and catalyst promoters such as S, Pb, and Bi were used. These catalyst promoters are known to increase the production yield of SWNT as well as alter the distribution of *d* [30,31,45].

Samples collected from the synthesis were analyzed using electron nanodiffraction technique on a modified VG microscopes HB-5 high resolution scanning transmission electron microscope (STEM) [33]. Convergent beam nanodiffraction was focused to nanometer sized diameter at the sample level. Field-emission guns, with a source diameter of about 4 nm, were used as the electron source for nanodiffraction. The STEM instrument used in this research has a cold-FEG source operated in ultra high vacuum, combined with condenser and objective lenses capable of forming a probe with diameter as small as 0.2 nm at the sample level. Two weak post-sample lenses have been added to the instrument in order to magnify the diffraction pattern over a wide range before observation. Diffraction patterns were recorded on a CCD camera. The STEM was operated at 100 kV, with a beam diameter of approximately 0.7 nm at the sample level. The nanodiffraction pattern can be recorded at any point along the STEM image by stopping the beam scan at that point. The expected diffraction pattern from an individual SWNT of a certain helicity can be derived using the general theory for diffraction from helical structures. A detailed description of the method can be found in References [33,35].

## 4. Conclusion

In summary, we have provided a comprehensive study of the helical angle of SWNT, as well as the distributions for specific regions within that sample. Experimental evidence provides strong support for a predominance of SWNT at *θ =* 30*°* (non-helical, armchair). Moreover, another peak at *θ =* 0*°* (non-helical, zigzag) conformation is also characteristic of the distribution. The distribution is important when characterizing bulk properties of as-produced SWNT, which is composed of nanotubes with different structures. We have also found that the helical angle distribution varies from region to region, indicating that the growth for SWNT is affected by fluctuations in the local environment. The PRN model, the screw dislocation theory, and theories based on the energetics and active sites of SWNT caps, provide a plausible explanation for the variations in helicity distributions in different regions.
